# Fluoroquinolone Persistence in *Escherichia coli* Requires DNA Repair despite Differing between Starving Populations

**DOI:** 10.3390/microorganisms10020286

**Published:** 2022-01-26

**Authors:** Annabel S. Lemma, Nashaly Soto-Echevarria, Mark P. Brynildsen

**Affiliations:** 1 Department of Chemical and Biological Engineering, Princeton University, Princeton, NJ 08544, USA; alemma@princeton.edu; 2 Department of Molecular Biology, Princeton University, Princeton, NJ 08544, USA; nashalye@princeton.edu

**Keywords:** nutrient deprivation, persisters, levofloxacin, moxifloxacin, heterotolerance

## Abstract

When faced with nutritional deprivation, bacteria undergo a range of metabolic, regulatory, and biosynthetic changes. Those adjustments, which can be specific or independent of the missing nutrient, often alter bacterial tolerance to antibiotics. Here, using fluoroquinolones, we quantified *Escherichia coli* persister levels in cultures experiencing starvation from a lack of carbon (C), nitrogen (N), phosphorous (P), or magnesium (Mg^2+^). Interestingly, persister levels varied significantly based on the type of starvation as well as fluoroquinolone used with N-starved populations exhibiting the highest persistence to levofloxacin, and P-starved populations exhibiting the highest persistence to moxifloxacin. However, regardless of the type of starvation or fluoroquinolone used, DNA repair was required by persisters, with ∆*recA* and ∆*recB* uniformly exhibiting the lowest persistence of the mutants assayed. These results suggest that while the type of starvation and fluoroquinolone will modulate the level of persistence, the importance of homologous recombination is consistently observed, which provides further support for efforts to target homologous recombination for anti-persister purposes.

## 1. Introduction

Nutrient limitation is a stress that bacteria can experience in a host [1,2,3]. For example, in the urinary tract, which is a common site of infections [4], urine constitutes a poor growth medium, since it lacks glucose, contains high concentrations of urea, and low levels of peptides and amino acids [1,5]. Throughout the body, phagocytes use nutrient deprivation as part of their antibacterial strategy for internalized pathogens [2,6,7]. Phagosomes have shown to be deficient in carbon, amino acids, nucleotides, and vitamins [2,7]. Further, when bacteria reside in biofilms or multicellular aggregates, nutrient gradients can arise depending on the chemical composition of the surroundings and metabolic activities of cells, which can produce starvation in some resident bacteria [8,9]. Importantly, the antibiotic tolerances of starved bacteria often exceed those of their growing counterparts, which contributes to the difficulties in treating nutrient-deprived bacteria effectively [10,11,12,13].

Although starvation, regardless of the type, has the common end-point of growth inhibition, the physiology of bacteria in growth arrest will depend on the limiting nutrient or nutrients [14,15,16,17]. For example, during glucose starvation the levels of regulatory metabolites cyclic AMP (cAMP) [18], guanosine 3′, 5′-bisphyrophosphate (ppGpp) [19], and phosphoenolpyruvate (PEP) increase, whereas fructose 1,6-biphospate (FBP) levels decrease [15,16]; and each regulates a different suite of activities [14]. During nitrogen (N) starvation, while an increase in ppGpp has been observed, cAMP and FBP levels appear unaffected, and PEP levels decline [15]. Heat production, CO_2_ generation, and O_2_ consumption differ between carbon (C)-, N-, and phosphate (P)-starved cultures [20], whereas glucose consumption varies between N-, P-, and Mg^2+^-starved populations, with Mg^2+^-starved cultures catabolizing glucose at the highest rate [16]. With such metabolic and regulatory differences, it is not surprising that the antibiotic susceptibilities of nutrient-depleted bacteria will depend on the missing nutrient. For example, with the aminoglycoside gentamicin, N- and P-starved cultures showed a notable level of susceptibility, whereas C-starved cultures were extremely tolerant [21]. In a study with nitrofurantoin, when stationary-phase bacteria were resuspended in saline, only C-supplied bacteria became sensitive to the antibiotic, whereas N-, P-, and Mg^2+^-supplied bacteria remained completely tolerant [22]. Collectively, these studies suggest that while growth inhibition in general increases the tolerances of bacteria to antibiotics, it may not do so in a uniform fashion.

Here, we examine how fluoroquinolone (FQ) persistence varies in growth-inhibited populations based on the identity of the missing nutrient. While most antibiotics are ineffective against non-growing bacteria, FQs, which target type II topoisomerases (DNA gyrase and topoisomerase IV), retain bactericidal activity [23,24,25]. During its catalytic cycle, topoisomerases produce transient breaks in DNA to modulate supercoiling, and consume two equivalents of ATP along the way [26,27,28,29]. Conceivably, differences in ATP could alter the activities of DNA gyrase and topoisomerase IV, which could impact sensitivity to FQs. Inspired by the physiological differences that have been observed between bacteria starved for different nutrients [14,15,16,17,18,19], we hypothesized that persistence to FQ will depend on the missing nutrient. Persistence is an extreme form of antibiotic tolerance that is exhibited by subpopulations in a bacterial culture [30,31,32], and most studies that have investigated FQ persistence in growth-inhibited populations have done so with stationary-phase cultures, which typically experience growth arrest due to exhaustion of carbon [23,24,25,33,34,35,36,37,38,39,40]. Here, we measured FQ persistence of *Escherichia coli* under long-term C-, N-, P-, or Mg^2^^+^-starvation, and examined the extent to which different DNA repair systems are used by FQ persisters while starving for different nutrients [23,24,34,35,37]. Specifically, we investigated *recA*, which is involved in homologous recombination (HR) and SOS induction [41]; *recB*, which is also involved in HR [42]; *lexA*, which represses the SOS response until its auto-cleavage is facilitated by RecA filaments that form on single-stranded DNA [43]; *uvrD*, which is involved in nucleotide excision repair and methyl-directed mismatch repair [44,45,46]; *ruvA*, which is involved in the resolution of Holliday junctions [47]; *recF*, which participates in recombinational repair of single-stranded DNA gaps left by the replisome [37]; and *recN*, which is involved in HR through its ability to facilitate contact between sister chromatids [48]. Interestingly, we found that persister levels vary based on both the type of starvation and FQ used, whereas the impacts of a variety of DNA repair systems on FQ persistence were largely invariant, with ∆*recA* and ∆*recB* constituting the most deleterious mutations. Collectively, these data illustrate that while the degree of persistence will depend on the nutritional environment and FQ used, the DNA repair systems used by persisters to survive FQ treatments are well-conserved.

## 2. Materials and Methods

### 2.1. Bacterial Strains and Plasmids

Strains used in this study are derived from *E. coli* MG1655 and summarized in Supplementary Table S1. ∆*recA*, ∆*recB*, ∆*uvrD*, ∆*ruvA*, ∆*recF*, and ∆*recN* mutations were transduced from the Keio collection [49] to *E. coli* MG1655 with the P1 phage method [50]. Kanamycin (KAN) resistance markers were removed using FLP recombinase expressed from pCP20. To construct the *lexA3* mutant*,* which is an uncleavable version of *lexA* [51], a Δ*malK* strain was first generated by P1 phage transduction from the Keio collection [49]. *malK* is located near *lexA* on the *E. coli* chromosome, and Δ*malK* cannot utilize maltose as a sole carbon source [52]. *lexA3* was transduced by P1 phage from CGSC 6550 [53] into *E. coli* MG1655 ∆*malK*, and colonies were selected on minimal maltose media plates. To verify the deletion of genes, PCR was run using external and internal primers listed in Supplementary Table S2. To verify the *lexA3* incorporation, the locus was amplified and sequencing was performed (Genewiz, South Plainfield, NJ, USA).

### 2.2. Chemicals and Media

All chemicals were obtained from Fisher Scientific (Pittsburgh, PA, USA) or Sigma Aldrich (Milwaukee, WI, USA). All media were made with distilled water purified with a Millipore Milli-Q lab water system (Burlington, MA) to a resistivity of 18.2 MΩ.cm. LB media used for pre-growth was made of 10 g/L tryptone, 5 g/L yeast extract, and 10 g/L NaCl dissolved in Milli-Q water, which was then sterilized by autoclaving. M9 minimal glucose media was used as the “complete media” in this study, from which C-starved, N-starved, P-starved, and Mg^2+^-starved versions were derived. The compositions of the different media are provided in Supplementary Table S3.

LB-agar plates were made with 25 g/L pre-mixed LB Miller broth and 15 g/L agar, which was autoclaved. For mutant selection, 50 μg/mL KAN was used. For *lexA3* mutant selection M9 minimal agar plates made with 6.78 g/L Na_2_HPO_4,_ 3 g/L KH_2_PO_4_, 1 g/L NH_4_Cl, 0.5 g/L NaCl, 0.011 g/L CaCl_2_, 0.24 g/L MgSO_4,_ and 10 mM maltose as the sole carbon source were used. For all wash steps, phosphate buffered saline (PBS) was prepared from autoclaved Milli-Q water and a 10X stock, followed by sterile-filtering (0.22 μM pore size). The 10X stock contained 98.9 g of powder (81% NaCl, 14% Na_2_HPO_4_, 3% KH_2_PO_4_, and 2% KCl by weight) in 1 L of Milli-Q water.

### 2.3. Culture Conditions

Cultures were first inoculated from −80 °C, 25% glycerol stocks into 2 mL of LB media in test tubes and incubated for 4 h at 37 °C with shaking (250 r.p.m.). After 4 h, those pre-growth cultures were diluted 100-fold in M9 media with 10 mM glucose and incubated at 37 °C with shaking (250 r.p.m.) overnight for 16 h. The 16 h overnight cultures were then diluted to an OD_600_ of 0.01 in 25 mL of M9 media with 10 mM glucose in 250 mL baffled flasks and grown to an OD_600_ of 0.2 at 37 °C. At OD_600_ of 0.2, 500 µL of the exponential-phase cultures was washed in the respective starvation media by centrifuging samples at 21,130 r.c.f. for 3 min, removing 450 µL of the supernatant, and then resuspending the cell pellet in 450 µL of the starvation media. This wash step was repeated two more times (total of three washes). For the growth assays, the samples were then diluted 10-fold to an OD_600_ of 0.02 into 25 mL of starvation media or M9 media. For the persister assays, the starvation samples were diluted 10-fold to an OD_600_ of 0.02 into 25 mL of starvation media and incubated for 16 h, whereas the M9 control samples were diluted 100,000-fold and incubated for 16 h to achieve an OD_600_ of ~0.02. Supplementary Figure S1 provides a schematic of the culturing conditions used in this study.

### 2.4. Growth Assay

Once cultures were inoculated into starvation media, OD_600_ was measured hourly with a Synergy H1 Hybrid Multi-Mode Microplate Reader (Agilent Technologies, Santa Clara, CA, USA) using 300 µL samples in flat-bottom 96-well plates. When necessary, cultures were diluted with similar media to ensure that OD_600_ readings were in the linear range of the spectrophotometer (0.01 to 0.4). To assess whether single-nutrient starvation conditions had been achieved, after 16 h of incubation in nutrient-starved media, missing nutrients or autoclaved water (control) were added to starved cultures and incubated for an additional 16 h, at which point OD_600_ measurements were taken.

### 2.5. Minimum Inhibitory Concentration Assay

Cultures were prepared by inoculating 25% glycerol stocks in 2 mL of LB media. After 16 h of incubation at 37 °C with shaking (250 r.p.m.), cultures were diluted to 10^5^ CFU/mL in 10 mL of Mueller–Hilton Broth (MHB). Antibiotic stock solutions (2 μg/mL) were prepared for both levofloxacin (LEVO) and moxifloxacin (MOXI), and 2-fold serial dilutions were performed in 75 µL of MHB in flat-bottom 96-well plates. Each well was then inoculated with 75 µL of diluted culture. An antibiotic-free well and an MHB-only well served as controls, respectively. Plates were covered with Breathe-Easy sealing membranes and incubated at 37 °C without shaking. After 20 h of incubation, OD_600_ were measured and when necessary samples were diluted 10-fold in MHB.

### 2.6. Persister Assay

After 16 h of incubation in starvation media, cultures were treated with 5 µg/mL of MOXI, LEVO or autoclaved Milli-Q water (MOXI MIC: 0.125 μg/mL, LEVO MIC: 0.0625 μg/mL, Supplementary Figure S2). Before addition of the antibiotic (*t* = 0) and at 1, 3 and 5 h, after the addition of the antibiotic, 500 µL samples were removed. Those samples were washed three times with PBS by centrifugation at 21,130 r.c.f. for 3 min, removal of 450 µL of the supernatant, and then resuspending the cell pellet in 450 µL of PBS. After three washes, the samples were then centrifuged again, followed by removal of 400 µL of supernatant, and resuspension of the cell pellet in the remaining 100 µL of PBS. That 5-fold concentrated sample was then serially diluted in PBS, plated on LB-agar, and incubated at 37 °C for 16 h, after which CFUs were enumerated.

### 2.7. Statistical Analysis

Data points indicate the average of at least three biological replicates. The error bars indicate the standard errors of the means. Where indicated, *t*-tests with unequal variances or one-way ANOVA with post-hoc Tukey tests were conducted to assess significance among the different treatment conditions.

## 3. Results

### 3.1. Establishing Single-Nutrient Starved Culturing Conditions with Complete Media Controls

**Figure 1:**
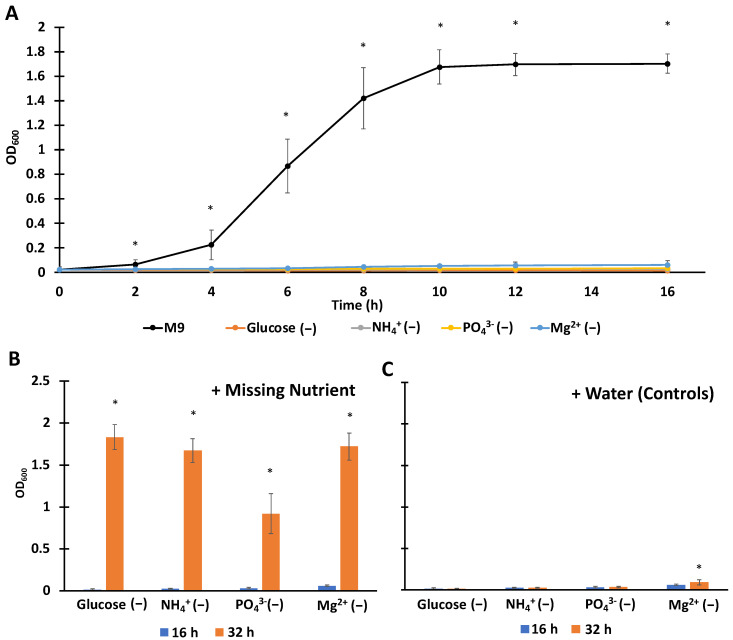
Establishment of culturing conditions for overnight starvation to individual nutrients. (**A**) Exponentially-growing wild-type (WT) cultures were inoculated into complete media or media deprived of carbon (C), nitrogen (N), phosphate (P), or magnesium (Mg^2+^) at an optical density at 600 nm (OD_600_) of 0.02. After inoculation, OD_600_ was measured at indicated time points, and all cultures in the nutrient-starved conditions exhibited growth inhibition. (**B**) After 16 h in nutrient-starved environments (0–16 h), the missing nutrients were supplied to cultures and incubated for an additional 16 h (16–32 h). Addition of the missing nutrient resulted in growth of all cultures, demonstrating single-nutrient limited growth. (**C**) Controls where autoclaved water was added in place of the missing nutrient and growth was monitored for an additional 16 h (16–32 h). Data points indicate the means of three biological replicates, and the error bars indicate the standard errors of the means. A schematic of the culturing procedure with respect to different assays is provided in Supplementary Figure S1. (**A**) One-way ANOVA with post-hoc Tukey tests were conducted to assess significance among the different treatment conditions. * Indicates significance with *p* < 0.05 with respect to the other treatment conditions at the same time point. (**B**,**C**) *t*-tests were conducted for significance analysis. * Indicates significance with *p* < 0.05 with respect to *t* = 0 h for the same treatment condition.

We sought to establish conditions where starvation occurred due to individual nutrients for a period of time equivalent to overnight, 16 h, along with a complete media control that was growing throughout that time. To accomplish that, we made variations of M9 media where specific components containing the nutrients of interest (C, N, P, Mg^2+^) were omitted, and for those usually provided as salts (N, P, Mg^2+^), their counter ions were provided as alternative salts (e.g., NaCl in place of NH_4_Cl) (Supplementary Table S3). As depicted in Figure 1A, all starvation media produced growth arrest. Cognizant that continued non-growing metabolism could render single-nutrient limited conditions deprived of additional nutrients, we assayed whether supplementation of individual missing nutrients could restore growth. As illustrated in Figure 1B, after 16 h of exposure to starvation media, supplementation with missing nutrients generated significant growth in all samples, whereas continued starvation extended growth arrest (Figure 1C). We note that the OD_600_ of the Mg^2+^-starved cultures supplemented with water increased slightly at 32 h compared to 16 h (Figure 1C), which we speculate reflects a modest difference in reductive division compared to other starvation scenarios. These data demonstrate that the cultures used here were starved for a single nutrient in all cases, even after 16 h incubation under starvation conditions.

### 3.2. FQ Persistence Levels Depend on the Type of Starvation and FQ Used

**Figure 2:**
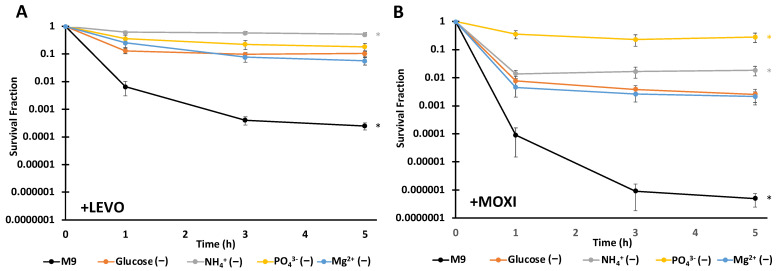
Fluoroquinolone (FQ) persistence levels depend on the type of nutrient starvation and antibiotic used. *E. coli* cultures that underwent 16 h of starvation for C, N, P, or Mg^2+^ and an exponentially-growing control in complete media (M9) were treated with 5 µg/mL levofloxacin (LEVO) (**A**) or 5 µg/mL moxifloxacin (MOXI) (**B**) for 5 h and survival was monitored. Before antibiotic treatment (at 0 h) and at 1, 3 and 5 h after antibiotic treatment, samples were obtained, washed in phosphate buffered saline (PBS) three times, and plated on LB-agar to enumerate colony forming units per milliliter (CFUs/mL). Data points indicate the means of five biological replicates, whereas error bars indicate the standard errors of those means. One-way ANOVA with post-hoc Tukey tests were conducted for each drug treatment to assess significance. (**A**) Complete media samples exhibited a significant difference with starvation samples at 1, 3, and 5 h time points. N-starved samples exhibited a significantly higher rate of survival from C-, P-, and Mg^2+^-starved samples at 3 and 5 h. (**B**) Complete media samples exhibited a significant difference with all starvation samples at 1, 3 and 5 h time points. P-starved samples exhibited a significantly higher rate of survival from C-, N-, and Mg^2+^-starved samples at 1, 3, and 5 h. N-starved samples exhibited a significantly higher rate of survival from C- and Mg^2+^-starved samples at 3 and 5 h time points. * Indicates a significance of *p* < 0.05.

Persistence assays were conducted with LEVO or MOXI on cultures incubated in starvation media for 16 h and a growing M9 control with the same approximate OD_600_ at the time of treatment. LEVO is the active isomer of ofloxacin (OFL), which is widely used in persistence studies [54]; and MOXI is a more recent FQ that has been identified as a more potent inhibitor of topoisomerase IV [55]. For both LEVO and MOXI, all nutrient-starved cultures exhibited significantly higher persister levels compared to cultures that were exponentially growing in M9 media (Figure 2). Under LEVO treatment, N-starved cultures exhibited the highest level of persistence with survival approaching 50%, which was significantly higher than C- and Mg^2+^-starved persister levels (Figure 2A). With MOXI, P-starvation exhibited the highest level of persistence with survival approaching 25%, which was significantly higher than C-, N-, and Mg^2+^-starved persister levels (Figure 2B). Under both FQ treatments, Mg^2+^ and C starvation gave similar levels of persistence. The observed differences in persistence among differently starved cultures suggest that persister levels depend on not just the type of starvation, but also the FQ used. Nutrient-starved cultures treated with water instead of FQ exhibited complete survival over the course of the experiments (Supplementary Figure S3).

### 3.3. FQ Persisters Depend on Similar DNA Repair Systems When Deprived of Different Nutrients

Previous studies have demonstrated the importance of different DNA repair enzymes to FQ persistence in non-growing cultures [23,24,34,35,37]. Given that the abundances of FQ persisters varied as a function of deprived nutrient and drug used, we assessed to what extent the DNA repair systems used by persisters in the different cultures were shared or distinct.

**Figure 3:**
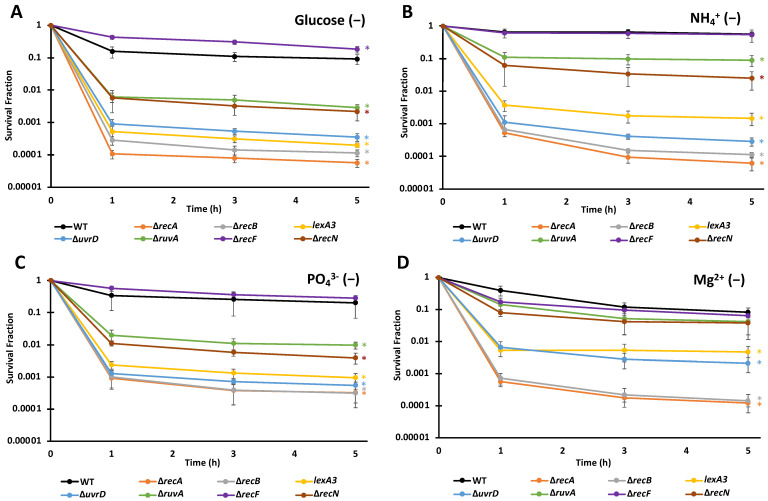
Importance of DNA repair machinery to LEVO persistence is largely invariant in non-growing populations starved for different nutrients. Persister levels in populations of ∆*recA*, ∆*recB*, *lexA3,* ∆*uvrD*, ∆*ruvA*, ∆*recF*, ∆*recN* and WT under the indicated starvation conditions were assessed with LEVO. Data points indicate the means of three biological replicates, whereas the error bars indicate the standard errors of those means. One-way ANOVA with post-hoc Tukey test were conducted for each starvation condition to assess significance. (**A**) Under C-starvation ∆*recF* exhibited significantly higher survival compared to WT at 1, 3, and 5 h. All other strains had significantly lower survival rates than WT at these time points. (**B**,**C**) Under N and P starvation all strains except ∆*recF* exhibited lower survival rates when compared to WT at 1, 3 and 5 h time points. (**D**) ∆*recA*, ∆*recB*, *lexA3,* ∆*uvrD* strains had a significantly lower survival rate compared to WT under Mg^2+^-starvation at 1, 3 and 5 h. Significance was identified as * *p* < 0.05.

**Figure 4:**
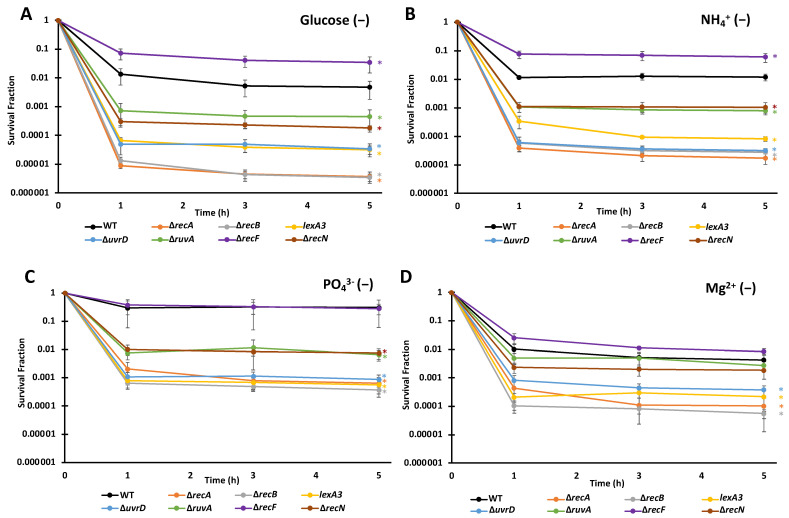
Importance of DNA repair machinery to MOXI persistence is largely invariant in non-growing populations starved for different nutrients. Persister levels in populations of ∆*recA*, ∆*recB*, *lexA3,* ∆*uvrD*, ∆*ruvA*, ∆*recF*, ∆*recN* and WT under the indicated starvation conditions were assessed with MOXI. Data points indicate the means of three biological replicates, whereas the error bars indicate the standard errors of those means. One-way ANOVA with post-hoc Tukey tests were conducted for each starvation conditions to assess significance. (**A**,**B**) Under C and N starvation ∆*recF* exhibited significantly higher survival compared to WT at 1, 3, and 5 h. All other strains had significantly lower survival rates than WT at these time points. (**C**) Under P starvation all strains except ∆*recF* exhibited significantly lower survival rates when compared to WT at 1, 3, and 5 h time points. (**D**) ∆*recA*, ∆*recB*, *lexA3,* ∆*uvrD* strains had a significantly lower survival rate compared to WT for Mg^2+^ starvation at 1, 3, and 5 h. Significance was identified as * *p* < 0.05.

Over the eight different combinations of starvation and FQ, we observed a similar ranking of importance of DNA repair systems for FQ persistence (Figure 3 and Figure 4, Supplementary Figures S4 and S5). Consistently, the mutants ∆*recA*, ∆*recB*, *lexA3*, ∆*uvrD*, ∆*ruvA*, and ∆*recN* were significantly lower than wild-type (WT), whereas ∆*recF* for some samples was significantly higher. We note that ∆*recF* has been shown before to increase persister levels in some conditions and that the effect depended on RecA, which suggested that the ability of RecF to load RecA onto ssDNA could be detrimental to survival following FQ treatment [37]. Deletions of *recA* and *recB* resulted in the largest declines in persister levels (typically ~1,000-fold reduction), followed by ∆*uvrD* mutants and an uncleavable *lexA* mutant (*lexA3*) (typically ~100-fold reduction), though for some scenarios these four mutants were indistinguishable (Figure 4C). Deletions of *ruvA* and *recN* constituted the next grouping, which yielded ~10-fold fewer persisters than WT; however, in environments devoid of Mg^2+^ their impacts were negligible (Figure 3D and Figure 4D). Interestingly, even for nutrient-deprived cultures with extremely high survival (N- and LEVO, P- and MOXI), DNA repair systems remained important, which suggested that even under conditions where FQs appear to largely lose their bactericidal activities, DNA damage occurs from treatment. 

## 4. Discussion

FQs are some of the few antibiotics that retain activity against non-growing bacteria [10,23,24], and their bactericidal activity derives from their binding to type II topoisomerases (DNA gyrase and topoisomerase IV in *E. coli*), which allows DNA cleavage but prevents ligation [26,56]. DNA gyrase is involved in replication and transcription by introducing negative supercoils in front of the replication fork and RNA polymerase [27,57,58]. Topoisomerase IV is involved in decatenating chromosomes at the end of replication, resolving DNA knots during recombination, and alleviating the over-winding of the double helix [26,59]. While nutrient starvation is known to stall DNA replication, transcription still continues at reduced rates [14,20,60,61], which suggests that the targets of FQs remain corruptible in nutrient-starved populations. Indeed, it has been observed that 90% or more stationary-phase *E. coli* can be killed by treatment with FQs [23,24,25,34,35,36,37]. 

Previous studies have investigated FQ persistence in stationary-phase *E.coli* populations [23,24,25,33,34,36,37,38,39], and though not explicitly characterized, most were likely growth inhibited due to lack of C [40]. However, many nutrients, when missing, produce growth arrest, and importantly, the physiology of growth-arrested bacteria will depend on the deprived nutrient [15,16,20,62]. Brown investigated persistence to ciprofloxacin (CIP) in *E. coli* populations having been starved of N for 20 min compared to N-replete controls [62]. Incipient N starvation increased CIP persistence compared to growing controls and roles for RelA and NtrC were identified; however, the role of DNA repair machinery was not examined [62]. Pontes and Groisman investigated tolerance to CIP in *Salmonella enterica* populations before and after exhaustion of limited supplies of Mg^2+^ [13]. One hour after Mg^2+^ exhaustion, CIP tolerance was observed to increase, and while the roles of (p)ppGpp and ATP in the phenomenon were evaluated, the importance of DNA repair was not assessed [13]. Studying persistence to a panel of antibiotics, including LEVO, Xu and colleagues found that 30 min after resuspension in saline, the tolerance of *Staphylococcus aureus* cultures increased dramatically [63]. Intriguingly, supplementation of saline with Mg^2+^ reduced the enhancement in LEVO tolerance through a pathway that involved ATP, whereas the role of DNA repair was not evaluated [63]. Wang and colleagues also used saline to examine antibiotic tolerance in starved cultures, where they observed a role for proton motive force in β-lactam susceptibility that was absent when cultures were treated with CIP [64]. In addition, Fung and colleagues conducted an expansive examination of nutrient availability and antibiotic tolerance that included OFL and C-, N-, and P-starved cultures [12]. Using 0.75 μg/mL of OFL on cultures that had been starved for 2 h, C starvation produced the highest enhancement in tolerance, although all three starvation conditions exhibited increased survival compared to a complete media control [12]. Importantly, RecA was found to be critical to OFL tolerance of cultures in growth-supporting media, after an overnight incubation, as well as those that were resuspended in MOPS base, which reflected a role for HR that was independent of the pre-growth and treatment environments [12]. 

In this study, we aimed to investigate longer periods of starvation (16 h) and compare a variety of types of nutrient limitation for their persistence phenotype and dependence on DNA repair (Table 1 and Table 2). Results indicated that regardless of the missing nutrient (C, N, P, or Mg^2+^) persistence increased in comparison to growing cultures, which was expected. We also observed that persister levels in growth-inhibited populations differed based on both the type of starvation and specific FQ used, which was unexpected and suggested that the environmental context even for non-growing bacteria matters for persistence to FQs. Here, we did not investigate the mechanisms of why different starvation types and FQs yielded quantitatively different persister levels, although those differences do represent an interesting area of study. Rather, inspired by previous studies that had shown that persistence to FQs in non-growing bacteria depended on the SOS response and DNA repair enzymes, we assayed several mutants defective in DNA repair and SOS induction [23,24,34,35,37,65]. Those genes included *recA*, which is a master mediator of HR and SOS induction [41,66]; *lexA*, which regulates the SOS response [43]; *recB*, which is involved in HR [42]; *ruvA*, which is involved in the final steps of recombination [47]; *uvrD*, which is involved in nucleotide excision repair [44,45]; *recF*, which helps load RecA onto single-stranded DNA [37]; and *recN,* which stimulates strand invasion by RecA in the repair of DSBs [48]. Previously, the impacts of those genes on persistence in non-growing populations were largely investigated in C-starved conditions [23,24,34,35,37,65] so we set out to assess their roles under deprivation of different nutrients: N, P, and Mg^2+^ in addition to C. Our results show that regardless of the type of starvation or the FQ used, the relative importance of DNA repair machinery and the SOS response remained largely invariant. We also observed that even in cultures that did not die appreciably from treatment, N-deprived cultures in the case of LEVO and P-deprived cultures in the case of MOXI, DNA repair was critical for persister survival.

The results presented here demonstrate that while the survival levels among different DNA repair mutants vary based on FQ and the starvation environment, the repair systems needed for FQ persistence in non-growing cultures are largely invariant. When this knowledge is combined with that from previous works where the importance of DNA repair machinery, such as *recA*, *recB*, *recG*, *lexA*, *ruvA*, *ruvB*, and *uvrD*, were established for FQ persistence in exponentially-growing cultures [37,67,68], it is straightforward to suggest that such systems can serve as universal targets for potentiation of FQs.

## Figures and Tables

**Table 1:** Survival fractions after 5 h of treatment with LEVO *.

Strain	Glucose (−)	NH_4_^+^ (-)	PO_4_^3−^ (−)	Mg ^2+^ (−)	M9
WT	9.26 × 10^−2^ (+/− 3.09 × 10^−2^)	5.58 × 10^−1^ (+/− 8.82 × 10^−2^)	2.02 × 10^−1^ (+/− 1.36 × 10^−1^)	8.28 × 10^−2^ (+/− 2.77 × 10^−2^)	2.50 × 10^−4^ (+/− 7.10 × 10^−5^)
∆*recA*	5.75 × 10^−5^ (+/− 1.63 × 10^−5^)	6.23 × 10^−5^ (+/− 2.61 × 10^−5^)	3.25 × 10^−4^ (+/− 1.69 × 10^−4^)	1.22 × 10^−4^ (+/− 2.74 × 10^−5^)	
∆*recB*	1.16 × 10^−4^ (+/− 2.84 × 10^−5^)	1.13 × 10^−4^ (+/− 1.75 × 10^−5^)	3.08 × 10^−4^ (+/− 2.00 × 10^−4^)	1.44 × 10^−4^ (+/− 8.21 × 10^−5^)	
*lexA3*	2.00 × 10^−4^ (+/− 2.92 × 10^−5^)	1.48 × 10^−3^ (+/− 6.00 × 10^−4^)	9.35 × 10^−4^ (+/− 3.45 × 10^−4^)	4.71 × 10^−3^ (+/− 2.41 × 10^−3^)	
∆*uvrD*	3.53 × 10^−4^ (+/− 9.52 × 10^−5^)	2.86 × 10^−4^ (+/− 8.02 × 10^−5^)	5.48 × 10^−4^ (+/− 1.37 × 10^−4^)	2.14 × 10^−3^ (+/− 1.04 × 10^−3^)	
∆*ruvA*	2.85 × 10^−3^ (+/− 7.19 × 10^−4^)	8.99 × 10^−2^ (+/− 3.30 × 10^−2^)	9.77 × 10^−3^ (+/− 2.37 × 10^−3^)	4.17 × 10^−2^ (+/− 3.03 × 10^−2^)	
∆*recF*	1.81 × 10^−1^ (+/− 4.20 × 10^−2^)	5.42 × 10^−1^ (+/− 2.22 × 10^−1^)	2.81 × 10^−1^ (+/− 6.08 × 10^−2^)	6.36 × 10^−2^ (+/− 1.65 × 10^−2^)	
∆*recN*	2.16 × 10^−3^ (+/− 1.04 × 10^−3^)	2.54 × 10^−2^ (+/− 1.46 × 10^−2^)	3.92 × 10^−3^ (+/− 1.42 × 10^−3^)	3.82 × 10^−2^ (+/− 2.18 × 10^−2^)	

* Mean (+/− standard error of the mean).

**Table 2:** Survival fractions after 5 h of treatment with MOXI *.

Strain	Glucose (−)	NH_4_^+^ (−)	PO_4_^3−^ (−)	Mg ^2+^ (−)	M9
WT	4.76 × 10^−3^ (+/− 2.98 × 10^−3^)	1.19 × 10^−2^ (+/− 3.11 × 10^−3^)	3.07 × 10^−1^ (+/− 2.47 × 10^−1^)	4.28 × 10^−3^ (+/− 2.49 × 10^−3^)	5.00 × 10^−7^ (+/− 2.51 × 10^−7^)
∆*recA*	3.71 × 10^−6^ (+/− 1.56 × 10^−6^)	1.73 × 10^−5^ (+/− 6.92 × 10^−6^)	6.46 × 10^−4^ (+/− 3.72 × 10^−4^)	1.03 × 10^−4^ (+/− 9.00 × 10^−5^)	
∆*recB*	3.48 × 10^−6^ (+/− 1.05 × 10^−6^)	2.75 × 10^−5^ (+/− 9.98 × 10^−6^)	3.70 × 10^−4^ (+/− 1.63 × 10^−4^)	5.54 × 10^−5^ (+/− 1.93 × 10^−5^)	
*lexA3*	3.20 × 10^−5^ (+/− 1.03 × 10^−5^)	8.09 × 10^−5^ (+/− 1.43 × 10^−5^)	5.65 × 10^−4^ (+/− 2.92 × 10^−4^)	2.19 × 10^−4^ (+/− 1.53 × 10^−4^)	
∆*uvrD*	3.42 × 10^−5^ (+/− 1.65 × 10^−5^)	3.14 × 10^−5^ (+/− 6.45 × 10^−6^)	8.72 × 10^−4^ (+/− 3.99 × 10^−4^)	3.80 × 10^−4^ (+/− 1.47 × 10^−4^)	
∆*ruvA*	4.54 × 10^−4^ (+/− 3.24 × 10^−4^)	7.89 × 10^−4^ (+/−1.53 × 10^−4^)	6.55 × 10^−3^ (+/− 2.60 × 10^−3^)	2.74 × 10^−3^ (+/− 1.04 × 10^−3^)	
∆*recF*	3.46 × 10^−2^ (+/− 1.96 × 10^−2^)	5.96 × 10^−2^ (+/− 2.02 × 10^−2^)	2.82 × 10^−1^ (+/− 1.14 × 10^−1^)	8.47 × 10^−3^ (+/− 2.20 × 10^−3^)	
∆*recN*	1.83 × 10^−4^ (+/− 2.94 × 10^−5^)	1.03 × 10^−3^ (+/− 4.66 × 10^−4^)	7.57 × 10^−3^ (+/− 3.02 × 10^−3^)	1.84 × 10^−3^ (+/− 9.36 × 10^−4^)	

* Mean (+/− standard error of the mean).

## Data Availability

The data presented in this study are available in the main text and Supplementary Material.

## Supplementary Materials

The following supporting information can be downloaded at: https://www.mdpi.com/article/10.3390/microorganisms10020286/s1. Figure S1: Schematic of culturing used with different assays. * dilution for M9 (complete media) samples was 100,000-fold for persister assays; however, it was 10-fold for growth assays so growth would be observable by OD600 measurements, Figure S2: MICs were determined using the microdilution protocol described in the Methods section with either (A) LEVO or (B) MOXI. Data points indicate the means of three biological replicates and the error bars indicate the standard errors of the means, Figure S3: Water-treated controls for FQ persister assays. (A) Survival fraction and (B) raw CFU/mL data depicting water-treated controls after 16 h starvation for C-, N-, P- and Mg^2+^-starved samples and a 16 h growing control in M9. Results show that the M9 growing control continued to grow, whereas starved samples did not. Data points indicate the means of three biological replicates and the error bars indicate the standard errors of the means, Figure S4: CFU/mL under LEVO treatment. Raw CFU/mL data for Figure 3. Data points indicate the means of three biological replicates and the error bars indicate the standard errors of the means, Figure S5: CFU/mL under MOXI treatment. Raw CFU/mL data for Figure 4. Data points indicate the means of three biological replicates and the error bars indicate the standard errors of the means, Table S1: Bacterial strains and Plasmids, Table S2: DNA oligonucleotides, Table S3. Composition of M9 and different starvation media.
