# Supplementary material for: Fluoroquinolone Persistence in Escherichia coli Requires DNA Repair despite Differing between Starving Populations
Source: Microorganisms. 2022 Jan 26;10(2):286. doi: 10.3390/microorganisms10020286 (PMC8877308; doi:10.3390/microorganisms10020286)
Supplement: Supplementary file 1 [file microorganisms-10-00286-s001.zip › microorganisms-1509135-supplementary.pdf]

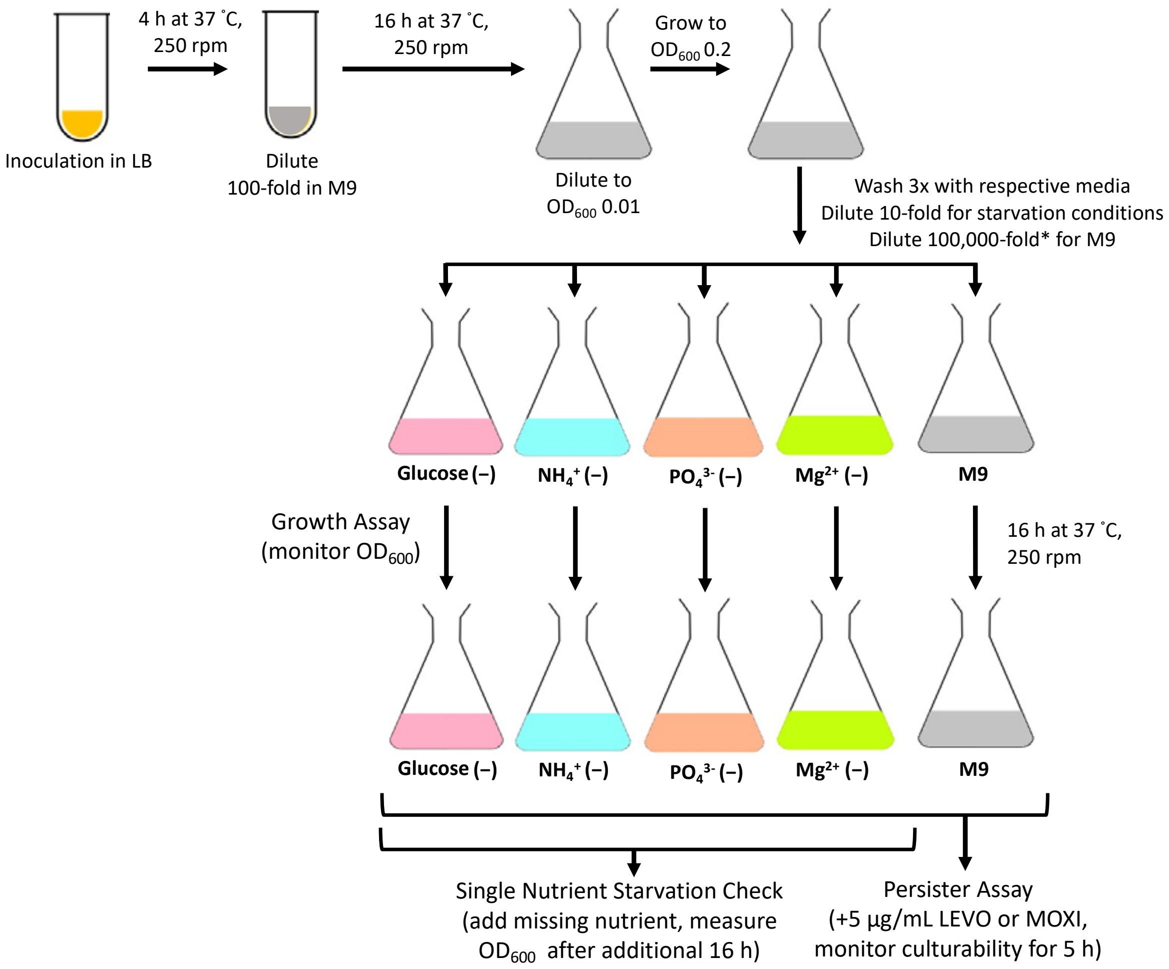

Supplementary Figure S1. Culturing Procedure. Schematic of culturing used with different assays. * dilution for M9 (complete media) samples was 100,000-fold for persister assays; however, it was 10-fold for growth assays so growth would be observable by OD_{600} measurements.

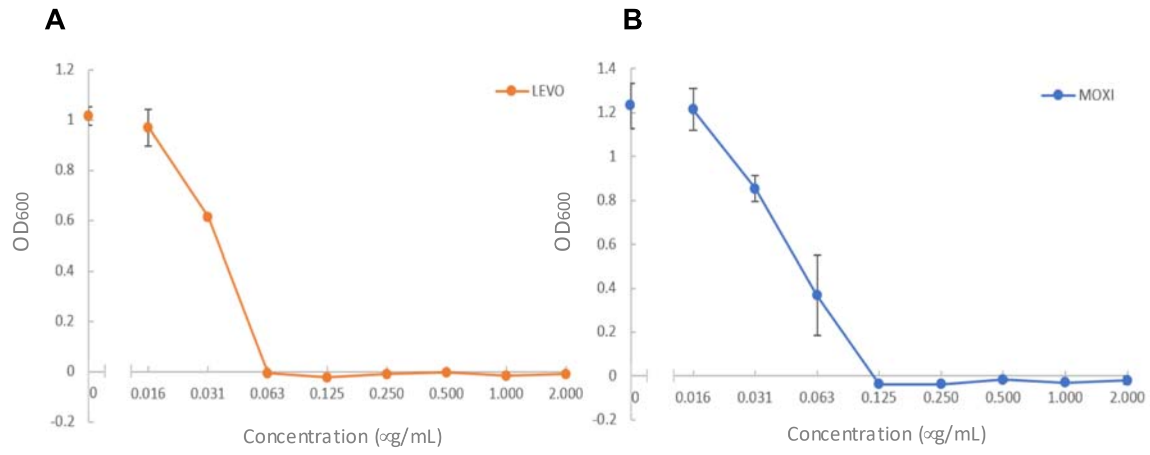

Supplementary Figure S2. Minimum inhibitory concentrations of *E. coli* MG1655. MICs were determined using the microdilution protocol described in the Methods section with either (A) LEVO or (B) MOXI. Data points indicate the means of three biological replicates and the error bars indicate the standard errors of the means.

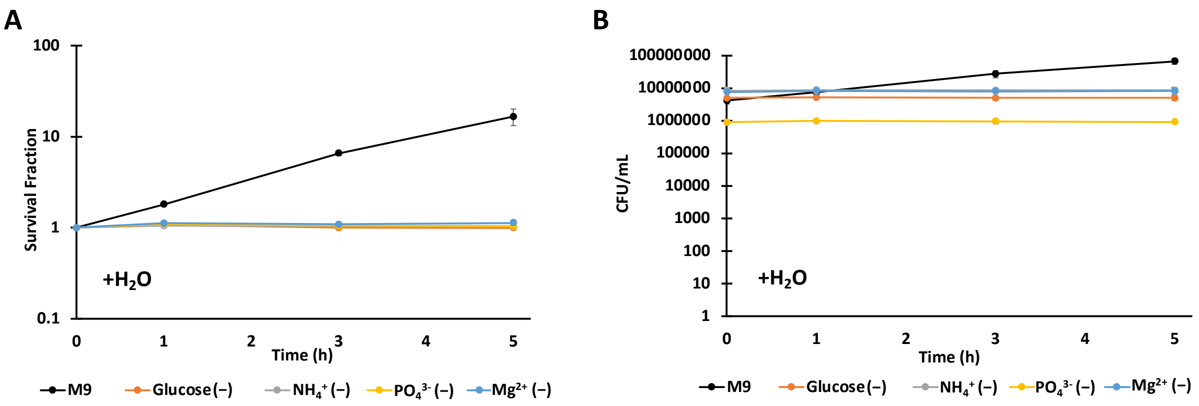

Supplementary Figure S3. Water-treated controls for FQ persister assays. (A) Survival fraction and (B) raw CFU/mL data depicting water-treated controls after 16 h starvation for C-, N-, P- and Mg^{2+}-starved samples and a 16 h growing control in M9. Results show that the M9 growing control continued to grow, whereas starved samples did not. Data points indicate the means of three biological replicates and the error bars indicate the standard errors of the means.

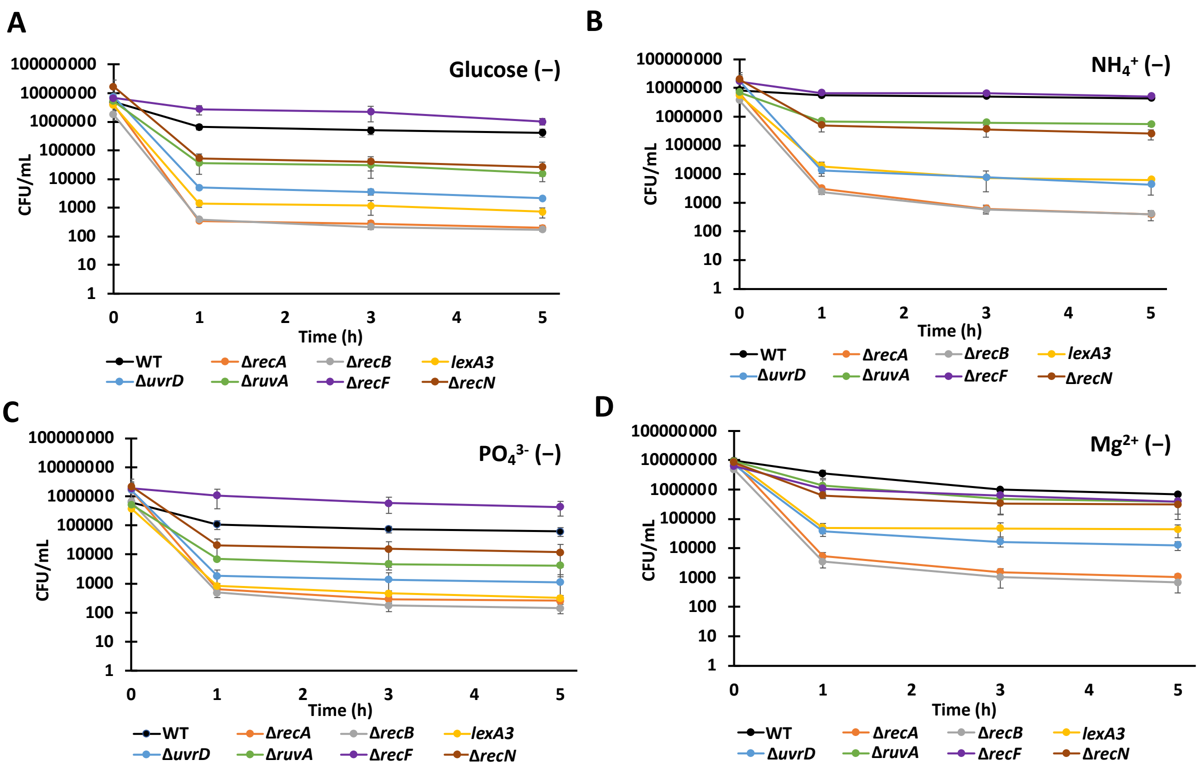

Supplementary Figure S4. CFU/mL under LEVO treatment. Raw CFU/mL data for Fig. 3. Data points indicate the means of three biological replicates and the error bars indicate the standard errors of the means.

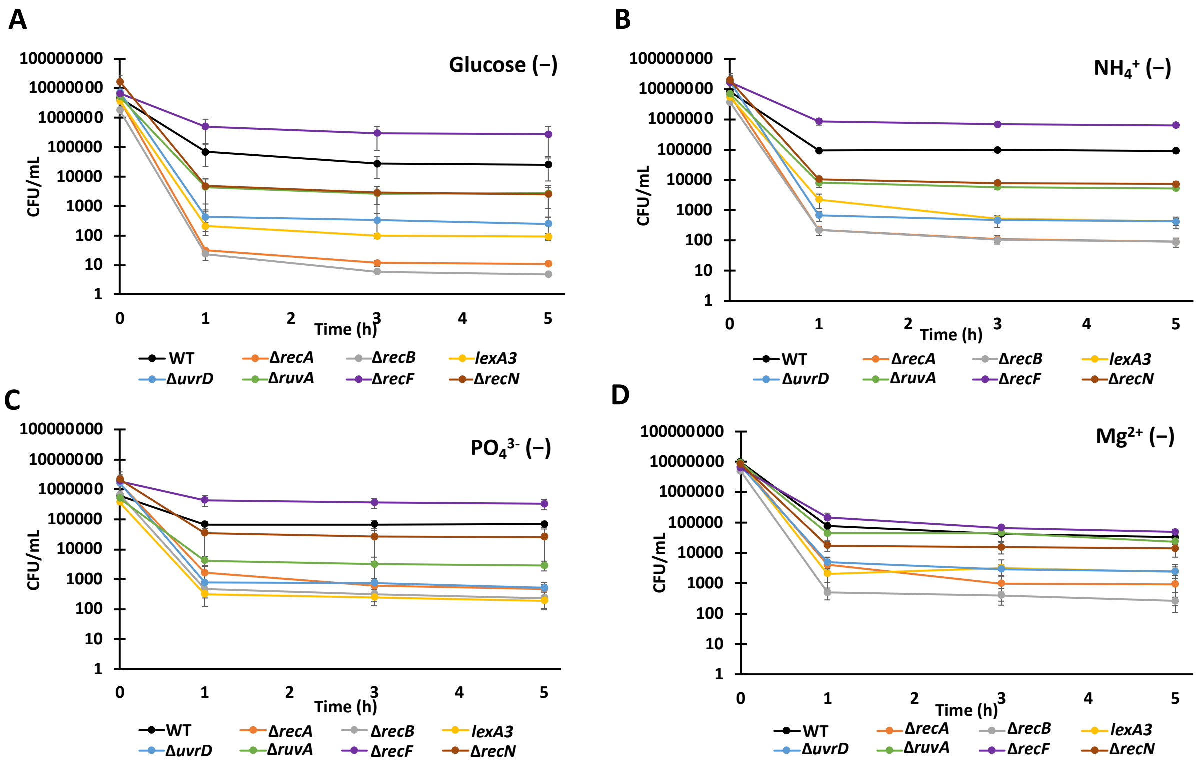

Supplementary Figure S5. CFU/mL under MOXI treatment. Raw CFU/mL data for Fig. 4. Data points indicate the means of three biological replicates and the error bars indicate the standard errors of the means.

Supplementary Table S1. Bacterial strains and Plasmids

Strain/ plasmid	Relevant genotype	Source	Description
MG1655	F-, λ-, *ilvG-*, *rfb-50, rph-1*	[1]	
Δ*recA*	MG1655 Δ*recA*	This work	Deletion of *recA* by P1 transduction of the corresponding deletion mutation from the Keio collection followed by curing the *kanR* resistance marker using pCP20
Δ*recB*	MG1655 Δ*recB*	This work	Deletion of *recB* by P1 transduction of the corresponding deletion mutation from the Keio collection followed by curing the *kanR* resistance marker using pCP20
Δ*uvrD*	MG1655 Δ*uvrD*	This work	Deletion of *uvrD* by P1 transduction of the corresponding deletion mutation from the Keio collection followed by curing the *kanR* resistance marker using pCP20
Δ*ruvA*	MG1655 Δ*ruvA*	This work	Deletion of *ruvA* by P1 transduction of the corresponding deletion mutation from the Keio collection followed by curing the *kanR* resistance marker using pCP20
Δ*recN*	MG1655 Δ*recN*	This work	Deletion of *recN* by P1 transduction of the corresponding deletion mutation from the Keio collection followed by curing the *kanR* resistance marker using pCP20
Δ*recF*	MG1655 Δ*recF*	This work	Deletion of *recF* by P1 transduction of the corresponding deletion mutation from the Keio collection followed by curing the

			kanR resistance marker using pCP20
Δ*malK*	MG1655 Δ*malK*	This work	Deletion of *malK* by P1 transduction of the corresponding deletion mutation from the Keio collection followed by curing the *kanR* resistance marker using pCP20
lexA3	MG1655 *lexA3*	This work	Integration of *lexA3* mutation by P1 transduction from CGSC 6550 into Δ*malK* and selection on minimal maltose plates
pCP20	AmpR and CmR, temperature sensitive replication (repA101ts), FLP recombinase gene, FLP	[2,3]	Cloning plasmid for curing FRT-flanked resistance markers

Supplementary Table S2. DNA oligonucleotides

**Primers used in chromosomal perturbations**		
**Primer name**	**Sequence**	**Description**
recA_ext_fwd	CTGGTTTGCTTTTGCCACTG	Forward primer external to *recA* used with *kanR* reverse or recA_ext_rev primers for cPCR check
recA_ext_rev	AATACGCGCAGGTCCATAAC	Reverse primer external to *recA* used with recA_ext_fwd primer for cPCR check
recA_int_fwd	TGGAAACCATCTCTACCGGTTC	Forward primer internal to *recA* used with *recA* internal reverse primer for cPCR check
recA_int_rev	GACGAACAGAGGCGTAGAAT	Reverse primer internal to *recA* used with *recA* internal forward primer for cPCR check
recB_ext_fwd	AACGGGAAAGCCGAATATGTACAC	Forward primer external to *recB* used with *kanR* reverse or

		recB_ext_rev primers for cPCR check
recB_ext_rev	GTTGCGCTACCTGGTCGT	Reverse primer external to *recB* used with recB_ext_fwd primer for cPCR check
recB_int_fwd	GCGGAAGATCTGCGTTTGCT	Forward primer internal to *recB* used with *recB* internal reverse primer for cPCR check
recB_int_rev	TCATAGCGGTGTGCCTGCAT	Reverse primer internal to *recB* used with *recB* internal forward primer for cPCR check
malK_ext_fwd	GCCAGGGGGTGGAGGATTTAAGC	Forward primer external to *malK* used with *kanR* reverse primer for cPCR check
malK_int_fwd	CCGTCTGGCTGCGGTAAATC	Forward primer internal to *malK*used with *malK* internal reverse primer for cPCR check
malK_int_rev	TGCCATCCTCACGGAACAGA	Reverse primer internal to *malK* used with *malK* internal forward primer for cPCR check
lexA_int_fwd	GTTAACGGCCAGGCAACAAG	Forward primer internal to *lexA* used with *lexA* internal reverse primer for sequencing check
lexA_int_rev	GCCCTTCAATGGTGAAGCTCT	Reverse primer internal to *lexA* used with *lexA* internal forward primer for sequencing check
uvrD_ext_fwd	TTACTGCCGCATCTGGAAAT	Forward primer external to *uvrD* used with *kanR* reverse or uvrD_ext_rev primers for cPCR check
uvrD_ext_rev	TACTGAAGATGGCGCAGATG	Reverse primer external to *uvrD* used with uvrD_ext_fwd primer for cPCR check
uvrD_int_for	TAATGACAAACAGCGCGAAG	Forward primer internal to *uvrD* used with *uvrD* internal reverse primer for cPCR check

uvrD_int_rev	CAGACGCCCGTTATTGTTTT	Reverse primer internal to *uvrD* used with *uvrD* internal forward primer for cPCR check
ruvA_ext_fwd	CATCGAGACACCTCGCAAGTT	Forward primer external to *ruvA* used with *kanR* reverse primer or ruvA_ext_rev primer for cPCR check
ruvA_ext_rev	AACAAATGATCGAGGGCATC	Reverse primer external to *ruvA* used with ruvA_ext_fwd primer for cPCR check
ruvA_int_fwd	AAGTGGGCGGCGTAGGCTAT	Forward primer internal to *ruvA* used with *ruvA* internal reverse primer for cPCR check
ruvA_int_rev	GCGGCTTGCTTCTTGTGGTT	Reverse primer internal to *ruvA* used with *ruvA* internal forward primer for cPCR check
recF_ext_fwd	GCGAAAACGTCCGCATGATG	Forward primer external to *recF* used with *kanR* reverse or recF_ext_rev primers for cPCR check
recF_ext_rev	TATACATACCCGGGCGCTTA	Reverse primer external to *recF* used with recF_ext_fwd primer for cPCR check
recF_int_fwd	TCCCTCACCCGCTTGTTGAT	Forward primer internal to *recF* used with *recF* internal reverse primer for cPCR check
recF_int_rev	TGATCGCGCTGACAAAGACC	Reverse primer internal to *recF* used with *recF* internal forward primer for cPCR check
recN_ext_fwd	CACCAAGCTCGGCTGGTCAA	Forward primer external to *recN* used with *kanR* reverse or recN_ext_rev primer for cPCR check
recN_ext_rev	GCAGCAGTCAGCGTTTTACA	Reverse primer external to *recN* used with recN_ext_fwd primer for cPCR check

recN_int_fwd	TGTTTGCTTCGTCGCGTGAT	Forward primer internal to *recN* used with *recN* internal reverse primer for cPCR check
recN_int_rev	CATTTTACGCGCCGTGATGA	Reverse primer internal to *recN* used with *recN* internal forward primer for cPCR check
KanR_rev	ATGATGGATACTTTCTCGGCAGGAG	Used with different external forward primers to confirm genomic locations of *kanR* cassettes

Supplementary Table S3. Composition of M9 and different starvation media

	Na_2HPO_4	KH_2PO_4	NH_4Cl	NaCl	$CaCl_2$	$MgSO_4$	Glucose	KCl	Na_2SO_4
M9	6.78g/L	3g/L	1g/L	0.5g/L	0.011g/L	0.24g/L	1.8g/L	-	-
Glucose (-)	6.78g/L	3g/L	1g/L	0.5g/L	0.011g/L	0.24g/L	-	-	-
NH_4^+ (-)	6.78g/L	3g/L	-	1.59g/L	0.011g/L	0.24g/L	1.8g/L	-	-
PO_4^{3-} (-)	-	-	1g/L	6.08g/L	0.011g/L	0.24g/L	1.8g/L	1.64g/L	-
Mg^{2+}(-)	6.78g/L	3g/L	1g/L	0.5g/L	0.011g/L	-	1.8g/L	-	0.28g/L

SUPPLEMENTARY MATERIAL REFERENCES

1. Kohanski, MA.; Dwyer, DJ.; Hayete, B.; Lawrence, CA.; Collins, JJ. A common mechanism of cellular death induced by bactericidal antibiotics. *Cell* **2007**. 130:797–810.
2. Cherepanov, PP.; Wackernagel, W. Gene disruption in *Escherichia coli*: TcR and KmR cassettes with the option of Flp-catalyzed excision of the antibiotic-resistance determinant. *Gene* **1995**. 158:9–14.
3. Datsenko, KA.; Wanner, BL. One-step inactivation of chromosomal genes in *Escherichia coli* K-12 using PCR products. *Proc Natl Acad Sci U S A* **2000**. 97:6640–6645.
